# Characteristics and Hospital Outcomes of Patients Admitted to the Intensive Care Unit Because of Haematological Malignancy—A Service Evaluation

**DOI:** 10.1002/jha2.70453

**Published:** 2026-11-05

**Authors:** Adam Harriman, Dhruv Parekh, James Hodson, Jonathan Weblin, Vicky MacBean

**Affiliations:** ^1^ Therapy Services University Hospitals Birmingham NHS Foundation Trust Birmingham UK; ^2^ Birmingham Acute Care Research Group Institute of Inflammation and Ageing University of Birmingham Birmingham UK; ^3^ University Hospitals Birmingham NHS Foundation Trust Birmingham UK; ^4^ Research Development and Innovation University Hospitals Birmingham NHS Foundation Trust Birmingham UK; ^5^ College of Health Medicine and Life Sciences Department of Health Sciences Brunel University of London London UK

**Keywords:** associative factors, haematological malignancy, haematology, service evaluation

## Abstract

**Introduction:**

Intensive care unit (ICU) admission due to haematological malignancy (HM) has previously been questioned due to high mortality. While mortality‐associated factors have been studied in HM patients to guide decision making and clinical practice, UK‐based research remains limited. This single‐centre retrospective service evaluation (SE) aimed to review characteristics and hospital outcomes of patients admitted to our ICU because of HM.

**Methods:**

Consecutive adults with HM‐related admissions to a multi‐specialty ICU between January 2022 and October 2024 were retrospectively identified. Data were extracted for patient characteristics, ICU interventions and hospital outcomes. Associations with ICU mortality rates were also assessed.

**Results:**

During the SE, there were 227 ICU admissions of HM patients, of which 168 were HM‐related and, hence, included in subsequent analysis. The most common reasons for admission were respiratory failure (36.3%) and sepsis (26.2%). ICU and in‐hospital mortality rates were 44.0% and 53.0%, respectively. ICU mortality rates increased significantly with APACHE II and SOFA scores (both *p* < 0.001), and in patients with leukaemia (*p* = 0.021), graft versus host disease (*p* = 0.001), treatment limitations (*p* < 0.001) or that were chemotherapy naïve (*p* = 0.044). In patients surviving to ICU discharge, 31.9% were unable to mobilise out of bed at this point. By hospital discharge, 94.9% of surviving patients were mobile; however, 25.3% required ongoing rehabilitation.

**Conclusion:**

This SE describes the characteristics, outcomes and mortality‐associated factors in patients with HM‐related admissions to our ICU, which may be useful in aiding clinician understanding and guiding future practice. Prospective, robust studies are needed to validate findings and explore future opportunities to enhance patient outcomes in this cohort.

**Trial Registration:**

The authors have confirmed clinical trial registration is not needed for this submission.

## 1 Introduction

Intensive care unit (ICU) survivorship in the United Kingdom has surpassed 85% [1], resulting in more patients with long‐term dysfunction and increased pressure on healthcare systems [2]. ICUs are an expensive and finite resource [3]; therefore, their use should be reserved for patients most likely to benefit [4]. Understanding the required resources, treatment burdens and expected outcomes for diseases is crucial for guiding clinician decision‐making and resource allocation [5]. Rehabilitation in the ICU after critical illness, commencing as early as possible, can improve outcomes and allow patients to regain their previous levels of function [6].

Haematological malignancies (HMs) are blood cancers stemming from dysfunctional blood cell production and function [7]. ICU mortality rates in patients with HM are higher than the general population, but have improved with advances in modern healthcare [8]. However, there is considerable global variability in reported ICU mortality rates, which range from 33% to 89.9% in single‐centre observational studies [9, 10, 11]. A UK national database review reported a rate of 43.1% [12] with smaller UK‐based observational studies evidencing ICU mortality rates of 33% and 29.4% respectively [13, 14]. Whilst ICU admission in this population is justified, consideration of illness trajectory and the burdensome nature of care should be given [15]. Within the United Kingdom, there are currently no admission guidelines or service standards for this cohort, meaning benchmarking and interpretation of service standards is challenging.

Identifying risk factors associated with mortality guides understanding of treatment success and futility [16]. Such factors are well researched for general ICU populations [17], with age, illness severity, mechanical ventilation (MV), sepsis and the degree of organ failure consistently being associated with ICU mortality [18]. However, evidence in HM patients is limited and inconsistent, possibly due to the unpredictability of terminal decline in HM stemming from the disease process and treatment‐specific factors [19]. This results in an unclear route from ICU admission to hospital survival [20]. The limited evidence in HM patients is also hampered by a lack of methodological rigour [21, 22].

The largest (*n* = 1011) ICU multi‐centre prospective study in HM patients found increasing age, poor functional performance status, illness severity, multi‐organ failure, MV, vasoactive medication and stem cell transplant (SCT) to be associated with increased ICU mortality, whereas disease remission and earlier ICU admission improved outcomes [23]. Worse outcomes in HM patients requiring MV or vasoactive medication is consistently observed across the literature [11, 24]. However, the association with SCT, illness severity scores, HM subgroup, neutropenia, infection and delayed ICU admission has been inconsistent and contradictory across other studies [21, 25, 26].

HM patients surviving ICU admission have also demonstrated moderate‐to‐severe frailty up to 12 months after discharge, including higher functional and psychological morbidity compared to general ICU populations. One study reported 60% of survivors had their candidacy for ongoing cancer treatment modified or removed due to poor health status [27]. Decisions regarding candidacy for ICU admission should also consider the long‐term functional outcomes, quality of life (QoL) and eligibility for ongoing treatment of survivors [28].

Clearer information on patient and hospital outcomes supports ICU and physiotherapy decision‐making, promoting judicious use of finite resources and optimising physiotherapy interventions through improved understanding of likely rehabilitation potential and trajectory [29]. As a result, limited physiotherapy provision can be redistributed towards patients most likely to benefit, as enhanced rehabilitation may reduce long‐term frailty and increase access to continued HM treatment [5, 28]. Improved insight into individuals’ rehabilitation potential may also streamline care pathways, strengthen continuity of care, and facilitate discharge planning, thereby reducing readmission rates and length of stay (LOS) [30, 31].

We aimed to complete a service evaluation (SE) to identify clinical characteristics of patients with HM‐related ICU admissions, describe their ICU and hospital outcomes and analyse clinical factors associated with mortality with the purpose of guiding local practice and service improvement initiatives.

## 2 Methods

### 2.1 Setting

A single‐centre SE was undertaken at a large tertiary hospital in the West Midlands, United Kingdom, comprising a multi‐speciality, 100‐bed ICU and specialist regional haemato‐oncological services. The ICU has twice‐daily consultant ward rounds and weekday Physiotherapy, Occupational Therapy, Dietetic and Speech and Language Therapy input. Patients requiring ICU admission are identified by elevated standardised early warning scores and reviewed by ICU outreach, with admission decisions made by a senior registrar or consultant intensivist. All patients are screened by Physiotherapy at ICU admission, with further weekday input prioritised based on clinical need and risk [32]. Staff resources are insufficient to provide daily or weekend rehabilitation; therefore, physiotherapy intervention at the weekend is limited to respiratory care only. Structured physical rehabilitation and mobilisation practice at the centre is largely undertaken by physiotherapy staff, with routine daily repositioning and some incidental rehabilitation activities being undertaken by nursing staff.

### 2.2 Population

Consecutive adult (age ≥ 16 years) ICU admissions of patients with HM between 1st January 2022 and 31st October 2024 were identified from the local Intensive Care National Audit and Research Centre dataset (ICNARC). Patients registered on the NHS National Data Opt‐Out were excluded, to ensure regulatory compliance [33]. The electronic health records system (EHR) was reviewed to identify the reason for ICU admission; patients were excluded if their admission was not directly HM‐related as documented in their EHR (e.g., traumatic brain injury with a history of leukaemia, or those in complete remission). Patients with multiple ICU admissions were treated separately if they stemmed from discrete hospital stays; readmissions to ICU within the same hospital stay were combined into a single episode for analysis.

### 2.3 Data Extraction

Data for patient demographics, medical history, routinely collected clinical information and HM‐specific details at ICU admission were extracted from EHRs. Pre‐admission frailty was quantified using the Clinical Frailty Scale (CFS) [34], with illness severity measured by the Sequential Organ Failure Assessment (SOFA) [35] and Acute Physiology and Chronic Health Evaluation II (APACHE II) scores [36]; the latter was additionally converted to the estimated in‐hospital mortality rate (‘APACHE II probability’). The primary reason for admission, as recorded on the ICNARC database, was identified. Patients’ treatment history was reviewed, to identify those that had undergone Cellular and Gene Therapy (CGT) with either SCT or chimeric antigen receptor T‐cell (CAR‐T) therapy. Patients with adverse events from these treatments (such as graft vs. host disease [GvHD], immune effector cell‐associated neurotoxicity syndrome, cytokine release syndrome, tumour lysis syndrome or cytopenias) were also identified. However, due to limitations in categorisation from the ICNARC databases, detailed specifics of adverse events were not available beyond GvHD in SCT recipients and ‘adverse events’ in those undergoing CAR‐T therapy.

ICU‐specific factors included the incidence of ICU‐acquired infection (defined as positive microbiological sampling) and neutropenia (neutrophil count ≤ 0.5 × 10^9^/L). Patients with ‘treatment limitations’ were identified, defined as a medical decision to restrict specific intervention within 24 h of ICU admission. The level of organ failure was quantified as the number of individual organs (from a total of six) requiring support, as per ICNARC criteria:

- Respiratory: Ventilation via high‐flow nasal oxygen (HFNO), non‐invasive ventilation (NIV) or invasive MV.
- Cardiovascular: Vasoactive medication requirement.
- Gastrointestinal: Intestinal failure requiring parenteral nutrition.
- Renal: Dialysis for anuria, or electrolyte/lactate clearance.
- Liver: Coagulopathy management with or without acute hepatocellular failure.
- Neurological: Consciousness level depression causing airway compromise.

LOS was recorded in calendar days, and excluded patients who died prior to reaching the end of the interval (e.g., ICU LOS excluded patients who died in ICU). In patients surviving to hospital discharge up to 30th November 2024, dates of death were extracted from national mortality data. Mobility at ICU and hospital discharge was quantified using the Manchester Mobility Score (MMS), a seven‐point scale used to measure physical function, ranging from bed‐based exercises (one point) to active mobilisation more than 30 metres (seven points) [37]. Hospital discharge destination was classified as ‘home’ (patient's normal residence), either with or without ongoing community rehabilitation team input; inpatient rehabilitation (i.e., a secondary inpatient rehabilitation centre); care home/hospice; or an inter‐hospital transfer.

### 2.4 Registration and Ethics

This project was registered and approved locally as a retrospective SE (Clinical Audits and Research Management system no. 22498); hence, ethical approval was not required as per the Health Research Authority decision making tool and the associated defining research matrix. As there are no formal reporting guidelines for SEs, results are reported as per STROBE guidelines, as these were believed to be the most appropriate [38].

### 2.5 Statistical Methods

Associations with ICU mortality rates were assessed using Fisher's exact test for categorical variables and Mann–Whitney *U* tests for ordinal or continuous variables. To visualise trends across continuous variables, ICU mortality rates were additionally reported within subgroups. Univariable binary logistic regression models were also produced for selected ordinal and continuous variables, which included the variable of interest as a continuous covariate, and ICU mortality as the dependent variable. Survival post‐hospital discharge was quantified using a Kaplan–Meier curve.

All analyses were performed using IBM SPSS v29 (IBM Corp. Armonk, NY). Statistical significance was primarily assessed using a threshold of *p* < 0.05; however, to assess the impact of multiple comparisons, hypothesis tests which remained significant at the threshold defined by the Benjamini–Hochberg procedure (across all *N* = 32 hypothesis tests performed, with a false discovery rate of 5%) were also identified. Continuous variables are summarised as mean ± standard deviation (SD) where approximately normally distributed, or as median (interquartile range [IQR]) otherwise. Complete data were available for all variables; hence, no considerations for missing data were required. Cases were excluded from the analyses of variables which were not applicable (e.g., patients who died in hospital were excluded from discharge destination analysis).

## 3 Results

### 3.1 Cohort Characteristics

A total of 227 ICU admissions in patients with HM were identified. After excluding those registered with NHS National Data Opt‐Out (*n* = 11), 48 (22.2%) of remaining admissions were unrelated to HM, and were excluded. The remaining 168 HM‐related ICU admissions (in 166 patients) were included (Figure 1). The median (IQR) age at ICU admission was 61 (44–69) years, with the majority being male (61.3%); pre‐admission, 73.2% were deemed either ‘well’ or ‘fit’ by the CFS (Table 1). Illness severity at ICU admission was high, with mean APACHE II probabilities and SOFA scores of 47% ± 21% and 7 ± 3, respectively. Leukaemia was the most prevalent HM subgroup (48.2%), followed by lymphoma (38.1%) and myeloma (13.7%; Table 2). Almost all (94.6%) patients had received, or were currently receiving, chemotherapy at ICU admission. A total of 47.6% of patients had received CGT, comprising SCT in 31.0% and CAR‐T therapy in 17.3% (one patient received both); adverse events occurred in 40.4% and 65.5% of SCT‐ and CAR‐T‐treated patients, respectively.

**FIGURE 1:**
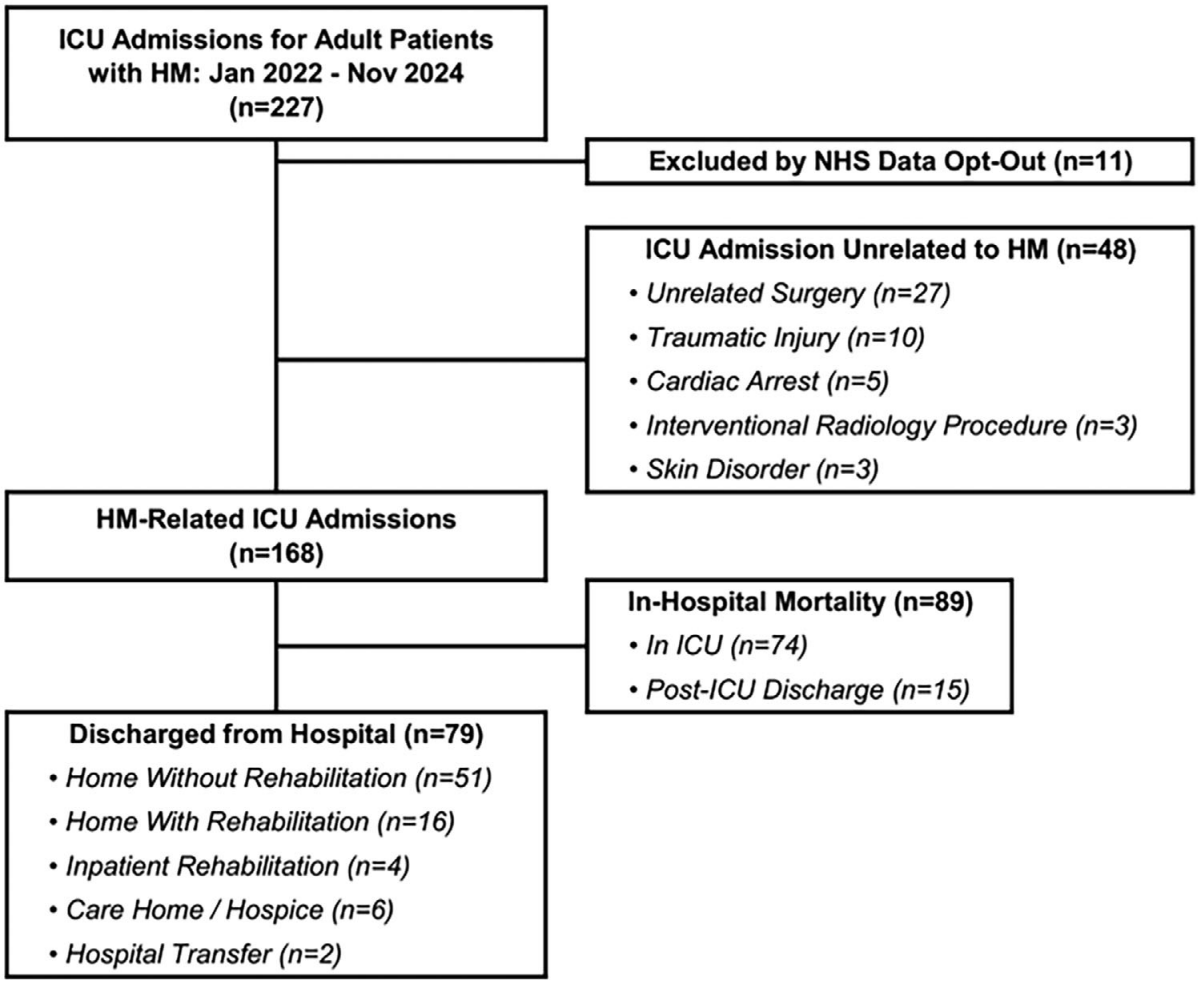
Flowchart diagram of inclusion/exclusions for admissions. HM: haematological malignancy; ICU: intensive care unit.

**TABLE 1:** Cohort characteristics at ICU admission.

	Statistic
Age (Years)	61 (44–69)
Male Sex	103 (61.3%)
BMI (kg/m^2^)	26.4 ± 6.2
Ethnicity	
White	132 (78.6%)
Asian	23 (13.7%)
Black	7 (4.2%)
Mixed/Other	6 (3.6%)
Clinical Frailty Scale	
1) Very Fit	1 (0.6%)
2) Well	31 (18.5%)
3) Managing Well	91 (54.2%)
4) Vulnerable	22 (13.1%)
5) Mildly Frail	17 (10.1%)
6) Moderately Frail	3 (1.8%)
7) Severely Frail	3 (1.8%)
APACHE II Score	24 ± 6
APACHE II Probability (%)	47 ± 21
SOFA Score	7 ± 3
**Past medical history**	
Diabetes Mellitus	
No	147 (87.5%)
Type 2	15 (8.9%)
Type 1	6 (3.6%)
Chronic renal failure	
No	154 (91.7%)
Yes—No Dialysis	10 (6.0%)
Yes—Dialysis	4 (2.4%)
Chronic respiratory disease	11 (6.5%)
Chronic liver disease	2 (1.2%)
Severe cardiovascular disease	2 (1.2%)

*Note*: Data are reported as mean ± standard deviation, median (interquartile range) or *N* (%), as applicable, and are based on *N* = 168, unless stated otherwise.

Abbreviations: APACHE, Acute Physiology and Chronic Health Evaluation; BMI, Body Mass Index; SOFA, Sequential Organ Failure Assessment.

**TABLE 2:** Details of haematological malignancy.

	Statistic
Haematological malignancy	
Acute myeloid leukaemia	53 (31.5%)
Acute lymphoblastic leukaemia	15 (8.9%)
Chronic lymphocytic leukaemia	7 (4.2%)
Chronic myelogenous leukaemia	6 (3.6%)
Non‐Hodgkin's lymphoma	58 (34.5%)
Hodgkin's lymphoma	6 (3.6%)
Multiple myeloma	23 (13.7%)
Disease status at ICU admission	
Partial remission	37 (22.0%)
Relapse	82 (48.8%)
New	49 (29.2%)
Current/Previous metastatic disease	24 (14.3%)
Current/Previous chemotherapy	159 (94.6%)
Current/Previous radiotherapy	8 (4.8%)
Prior steroid treatment	11 (6.5%)
History of stem cell transplant	
No	116 (69.0%)
Allograft	36 (21.4%)
Autograft	16 (9.5%)
Graft vs. Host Disease [*N* = 52]^a^	21 (40.4%)
History of CAR‐T therapy	29 (17.3%)
CAR‐T‐related adverse event	19 (65.5%)

*Note*: Data are reported as mean ± standard deviation, median (interquartile range) or *N* (%), as applicable, and are based on *N* = 168, unless stated otherwise.

Abbreviations: CAR‐T, Chimeric Antigen Receptor T‐cell; ICU, intensive care unit.

^a^ Patients with a history of SCT or CAR‐T therapy (as applicable) with a related adverse event as the cause of the ICU admission, or where this developed during the ICU stay.

SCT incidence was 31.0%, of whom 40.4% of patients developed GvHD. The median (IQR) time from hospital to ICU admission was 10 (2, 3, 4, 5, 6, 7, 8, 9, 10, 11, 12, 13, 14, 15, 16, 17) days, with most patients admitted due to respiratory failure (36.3%) or sepsis (26.2%; Table 3). Treatment limitations were set in 14.3% of cases, with 55.4% of patients receiving steroids and 53.0% developing neutropenia. No organ support was required in ICU for 10.7% of patients, with single‐ and multi‐organ failure in 30.4% and 58.9%; organ failure was most commonly respiratory (67.9%) or cardiovascular (67.3%).

**TABLE 3:** Details of ICU admission.

	Statistic
Hospital admission to ICU admission (days)	10 (2–17)
Primary reason for ICU admission	
Respiratory failure	61 (36.3%)
Sepsis/Septic shock	44 (26.2%)
<24 H Post‐CGT	16 (9.5%)
Neurological disorder	15 (8.9%)
Medical—Other	7 (4.2%)
Surgical procedure	7 (4.2%)
Cardiovascular failure	6 (3.6%)
Renal failure	5 (3.0%)
Tumour lysis syndrome	3 (1.8%)
Intestinal failure	2 (1.2%)
Dermatological condition	1 (0.6%)
Liver failure	1 (0.6%)
Acute steroids during ICU admission	93 (55.4%)
Neutropenia during ICU admission	89 (53.0%)
Treatment limitations^a^	24 (14.3%)
Respiratory failure	
No	54 (32.1%)
High flow nasal oxygen	34 (20.2%)
Non‐invasive ventilation	8 (4.8%)
Mechanical ventilation	72 (42.9%)
Cardiovascular failure	113 (67.3%)
Gastrointestinal failure	11 (6.5%)
Liver failure	18 (10.7%)
Neurological failure	6 (3.6%)
Renal failure	54 (32.1%)
Level of organ failure^b^	
0	18 (10.7%)
1	51 (30.4%)
2	47 (28.0%)
3	39 (23.2%)
4	11 (6.5%)
5	2 (1.2%)

*Note*: Data are reported as mean ± standard deviation, median (interquartile range) or *N* (%), as applicable, and are based on *N* = 168, unless stated otherwise.

Abbreviations: ICU, intensive care unit.

^a^ At or within 24 h of ICU admission.

^b^ The number of organs that failed during the ICU admission, considering a total of six: respiratory, cardiovascular, gastrointestinal, liver, neurological or renal.

The in‐hospital mortality rate was 53.0%, comprising 44.0% ICU deaths with a further 8.9% post‐ICU discharge (Table 4). Patients surviving ICU had a median (IQR) ICU LOS of 6 (3, 4, 5, 6, 7, 8, 9, 10) days, with 31.9% being unable to mobilise out of bed (MMS ≤ 2) and 53.2% able to step‐transfer or better (MMS ≥ 5) at this point. Patients surviving to hospital discharge had a median (IQR) LOS of 30 (19–51) days; 94.9% were able to mobilise (MMS ≥ 6) at this point, although 25.3% required ongoing rehabilitation post‐discharge. Over a median (IQR) of 9 (5, 6, 7, 8, 9, 10, 11, 12, 13, 14, 15, 16, 17, 18, 19, 20) months of post‐hospital discharge follow‐up, there were 19 deaths, yielding Kaplan–Meier estimated survival rates of 88%, 80%, and 72% at 90 days, six months and one‐year post‐hospital discharge, respectively (Figure 2).

**TABLE 4:** Patient outcomes.

	Statistic
ICU‐acquired infection	55 (32.7%)
In‐hospital mortality	
No	79 (47.0%)
In ICU	74 (44.0%)
Post‐ICU discharge^a^	15 (8.9%)
ICU LOS (days) [*N* = 94]^b^	6 (3–10)
MMS at ICU discharge [*N* = 94]^b^	
1 (Bed‐based exercises)	25 (26.6%)
2 (Sit on edge of bed)	5 (5.3%)
3 (Hoisted to chair)	3 (3.2%)
4 (Standing practice)	11 (11.7%)
5 (Step‐transfer to chair)	34 (36.2%)
6 (Mobilise < 30m)	12 (12.8%)
7 (Mobilise > 30m)	4 (4.3%)
Total hospital LOS (Days) [*N* = 79]^c^	30 (19–51)
MMS at hospital discharge [*N* = 79]^c^	
1 (Bed‐based exercises)	0 (0.0%)
2 (Sit on edge of bed)	1 (1.3%)
3 (Hoisted to chair)	2 (2.5%)
4 (Standing practice)	1 (1.3%)
5 (Step‐transfer to chair)	0 (0.0%)
6 (Mobilise < 30 m)	25 (31.6%)
7 (Mobilise > 30 m)	50 (63.3%)
Discharge destination [*N* = 79]^c^	
Home (no rehabilitation)	51 (64.6%)
Home with rehabilitation	16 (20.3%)
Inpatient rehabilitation	4 (5.1%)
Care Home/Hospice	6 (7.6%)
Hospital transfer	2 (2.5%)

*Note*: Data are reported as mean ± standard deviation, median (interquartile range) or *N* (%), as applicable, and are based on *N* = 168, unless stated otherwise.

Abbreviations: ICU, intensive care unit; LOS, length of stay; MMS, Manchester Mobility Score.

^a^ Death in hospital after ICU discharge.

^b^ In patients surviving to ICU discharge.

^c^ In patients surviving to hospital discharge.

**FIGURE 2:**
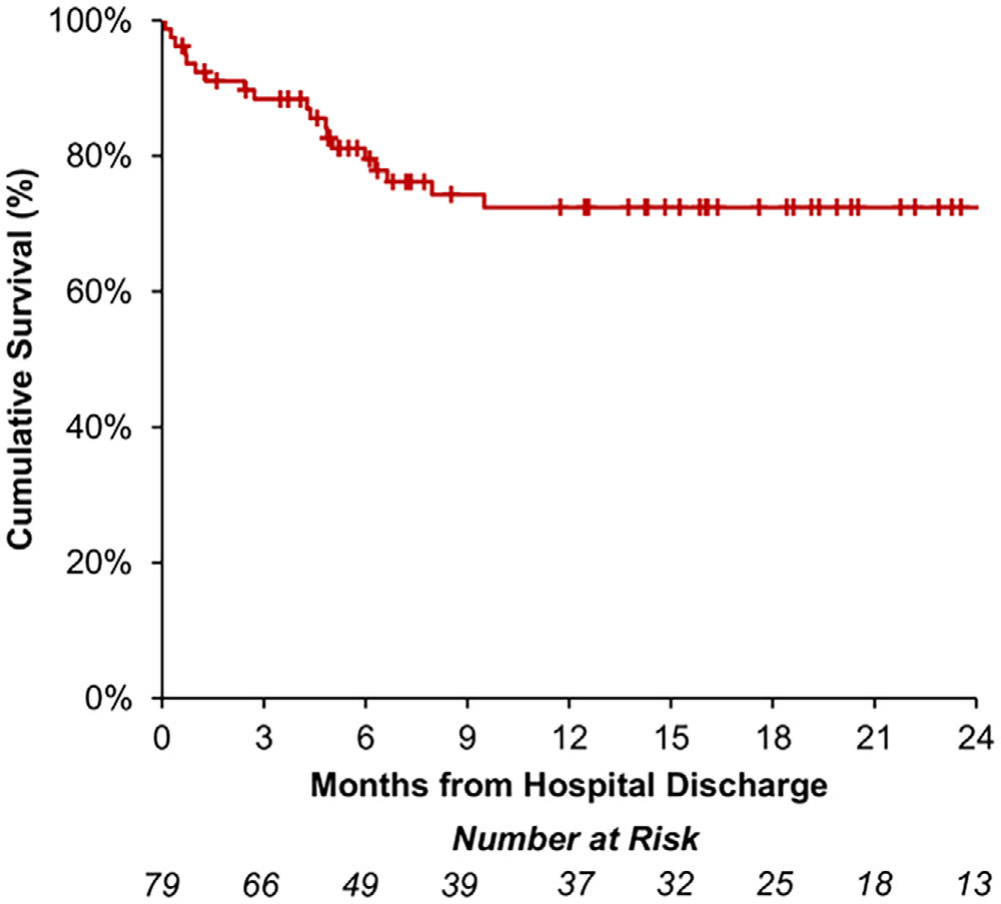
Kaplan–Meier curve of survival post‐hospital discharge. Analysis is based on the *N* = 79 patients who survived to hospital discharge. The plot is truncated at 24 months of follow‐up, due to declining numbers at risk.

### 3.2 Associations With ICU Mortality

ICU mortality was not found to differ significantly with patient age (*p* = 0.241), sex (*p* = 0.429), BMI (*p* = 0.813) or ethnicity (*p* = 1.000; Table 5a). However, ICU mortality rates were found to increase significantly with the APACHE II probability (*p* < 0.001; Figure 3a) and SOFA score (*p* < 0.001; Figure 3b); no significant association with CFS was observed (*p* = 0.063; Figure 3c). Of the disease‐related factors considered (Table 5b), ICU mortality rates were found to be significantly higher in patients with chronic renal failure (*p* = 0.047) and differed significantly by HM subgroup, ranging from 31% in lymphoma to 54% in leukaemia (*p* = 0.021). Chemotherapy‐naïve patients had significantly higher mortality than chemotherapy recipients (78% vs. 42%, *p* = 0.044). Since CGT‐related adverse events were only applicable to patients with a history of CGT, these variables were combined for analysis, which found ICU mortality rates to differ significantly across the five categories (*p* = 0.0001). Specifically, ICU mortality rates were lowest in patients undergoing either SCT or CAR‐T therapy without adverse events (26% and 10% respectively), and highest in patients with GvHD after SCT (70%).

**TABLE 5A:** Associations between patient characteristics and ICU mortality.

	ICU mortality rate	*p*‐Value
Age (years)^a^		0.241^a^
<45	40% (17/43)	
45–59	43% (15/35)	
60–69	50% (25/50)	
≥ 70	43% (17/40)	
Sex		0.429
Female	40% (26/65)	
Male	47% (48/103)	
BMI (kg/m^2^)^a^		0.813^a^
< 25	46% (36/79)	
25–29.9	45% (25/56)	
30–34.9	50% (7/14)	
≥ 35	32% (6/19)	
Ethnicity		1.000
White	44% (58/132)	
Non‐White	44% (16/36)	
Clinical Frailty Scale^a^		0.063^a^
1–2	41% (13/32)	
3	37% (34/91)	
4	68% (15/22)	
5–7	52% (12/23)	
APACHE II Probability (%)^a^, ^b^		**< 0.001** *a
< 30%	24% (9/37)	
30%–44%	30% (13/44)	
45%–59%	51% (22/43)	
≥ 60%	68% (30/44)	
SOFA Score^a^		**< 0.001** *a
0–4	19% (7/37)	
5–7	36% (20/55)	
8–9	54% (19/35)	
≥ 10	68% (28/41)	

*Note*: All variables were assessed at the time of ICU admission. Data are reported as the ICU mortality rate within each subgroup, with *p*‐values from Fisher's exact tests, unless stated otherwise. Bold *p*‐values are significant at *p* < 0.05.

Abbreviations: APACHE, Acute Physiology and Chronic Health Evaluation; BMI, Body Mass Index; ICU, intensive care unit, SOFA, Sequential Organ Failure Assessment.

^a^ Ordinal/continuous variables were grouped into categories within which the ICU mortality rates are reported; *p*‐values are from Mann–Whitney *U* tests on the ungrouped data (e.g., comparing the average age between patients that did vs. did not die in ICU).

^b^ The APACHE II can be summarised based on a raw score or predicted probability; since these are highly correlated (Spearman's rho: 0.9), only the latter was considered, to prevent duplication.

* Remains significant after adjustment for multiple comparisons using the Benjamini–Hochberg procedure (for *N* = 32 tests and a false discovery rate of 0.05).

**FIGURE 3:**
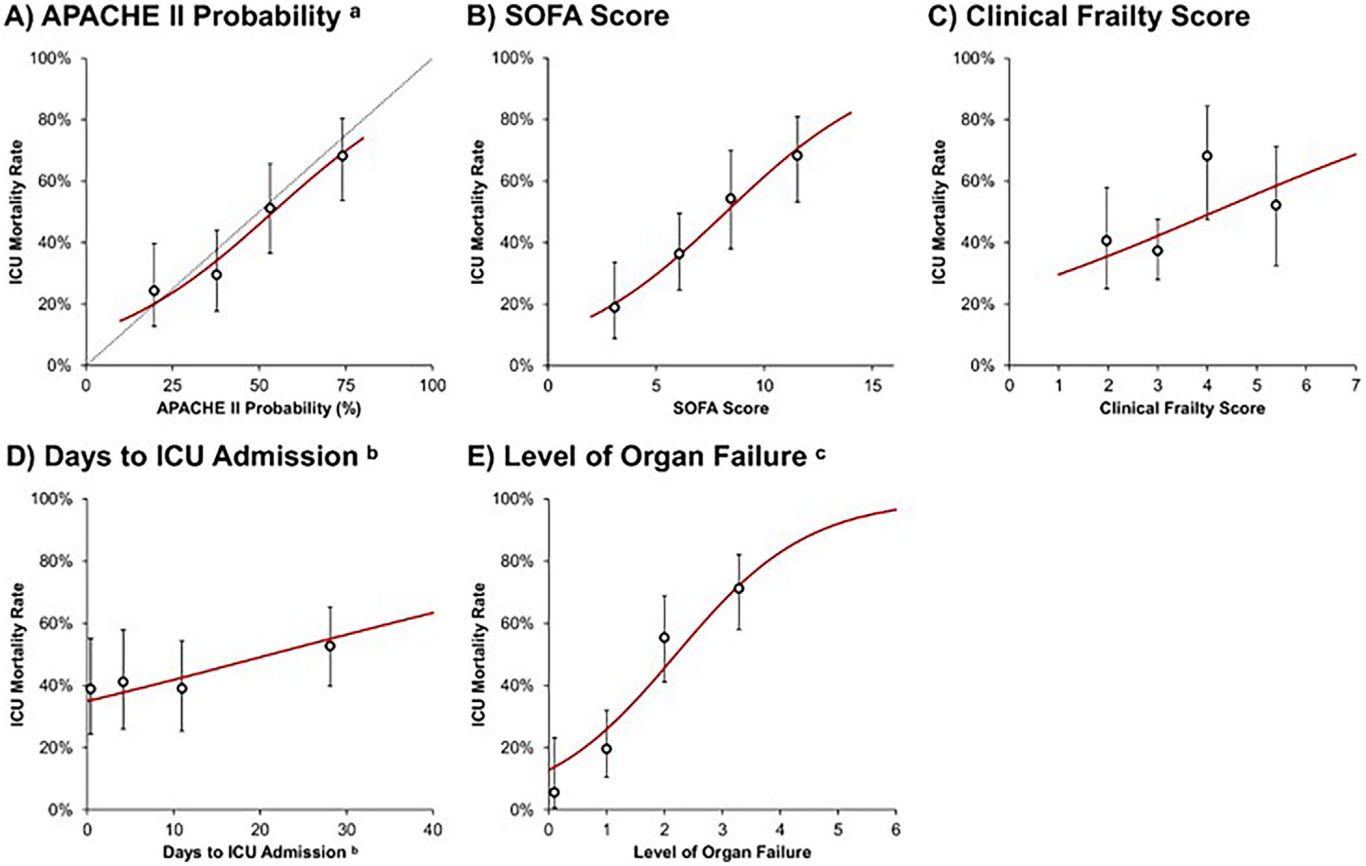
Associations with ICU mortality. Points represent the ICU mortality rates within subgroups, as per **Table** **5a**–d, and are plotted at the mean value within each subgroup; whiskers represent 95% CIs. Trend lines are from univariable binary logistic regression models on the ungrouped data, with each variable as a continuous covariate; the associated odds ratios and 95% CIs are tabulated in the final panel. ^a^A broken line is plotted at *y* = *x*, representing the expected trajectory of the trend line if the mortality rates predicted by the APACHE II were accurate; the odds ratio is reported per 10 percentage point (pp) increase. ^b^The number of days from hospital admission to ICU admission; the odds ratio is reported per seven‐day increase. ^c^The point for a level of organ failure of 0 is plotted at a value of 0.1, for visibility. 95% CI: 95% confidence interval; APACHE, Acute Physiology and Chronic Health Evaluation; ICU, intensive care unit; pp, percentage point; SOFA, sequential organ failure assessment.

**TABLE 5B:** Associations between past medical history/haematological malignancy and ICU mortality.

	ICU mortality rate	*p*‐Value
Diabetes mellitus		0.484
No	43% (63/147)	
Type 1 or 2	52% (11/21)	
Chronic renal failure		**0.047**
No	42% (64/154)	
Yes (±Dialysis)	71% (10/14)	
Chronic respiratory disease		0.539
No	43% (68/157)	
Yes	55% (6/11)	
Chronic liver disease		1.000
No	44% (73/166)	
Yes	50% (1/2)	
Severe cardiovascular disease		1.000
No	44% (73/166)	
Yes	50% (1/2)	
Haematological malignancy		**0.021**
Leukaemia	54% (44/81)	
Lymphoma	31% (20/64)	
Multiple myeloma	43% (10/23)	
Disease status		0.097
Partial remission	38% (14/37)	
Relapse	39% (32/82)	
New	57% (28/49)	
Current/Previous metastatic disease		0.828
No	44% (64/144)	
Yes	42% (10/24)	
Current/Previous chemotherapy		**0.044**
No	78% (7/9)	
Yes	42% (67/159)	
Current/Previous radiotherapy		0.079
No	46% (73/160)	
Yes	13% (1/8)	
Prior steroid treatment		0.539
No	43% (68/157)	
Yes	55% (6/11)	
History of CGT/Adverse events^a^ No SCT—No adverse event CAR‐T—No adverse event SCT—With GvHD CAR‐T—With adverse event	51% (45/88) 26% (8/31) 10% (1/10) 70% (14/20) 32% (6/19)	0.001*

*Note*: Data are reported as the ICU mortality rate within each subgroup, with *p*‐values from Fisher's exact tests. Bold *p*‐values are significant at *p* < 0.05.

Abbreviations: CAR‐T, Chimeric Antigen Receptor T‐cell; CGT, cellular and gene therapy; GvHD, Graft versus host disease; ICU, intensive care unit; SCT, stem cell transplant.

^a^History of SCT and CAR‐T, and occurrence of the associated adverse events were combined into a composite variable for analysis; one patient with a history of both SCT and CAR‐T was included the CAR‐T subgroup for analysis, due to presenting with a CAR‐T‐related adverse event.

* Remains significant after adjustment for multiple comparisons using the Benjamini–Hochberg procedure (for *N* = 32 tests and a false discovery rate of 0.05).

Analysis of ICU‐related variables found significantly higher ICU mortality rates in patients with a longer time to ICU admission (*p* = 0.048; Table 5c; Figure 3d). ICU mortality rates also differed significantly by reason for ICU admission (*p* = 0.040), ranging from 25% in those experiencing HM‐related treatment complications, to 56% following respiratory failure. Treatment limitations within 24 h of ICU admission were associated with significantly higher ICU mortality rates (88% vs. 37%, *p* < 0.001). ICU mortality rates increased significantly with the level of organ failure (*p* < 0.001; Figure 3e), with analyses of individual organs finding renal (*p* < 0.001), respiratory (*p* < 0.001) and cardiovascular (*p* = 0.020) failure to be most strongly associated (Table 5d).

**TABLE 5C:** Associations between ICU‐related variables and ICU mortality.

	ICU mortality rate	*p*‐Value
Hospital Admission to ICU Admission (Days)^a^		**0.048** ^a^
0–1	39% (14/36)	
2–7	41% (14/34)	
8–14	39% (16/41)	
> 14	53% (30/57)	
Reason for ICU admission		**0.040**
Respiratory failure	56% (34/61)	
Sepsis/Septic shock	30% (13/44)	
Complications of treatment	25% (4/16)	
Neurological disorder	47% (7/15)	
Others	50% (16/32)	
Acute steroids during ICU admission		0.354
No	40% (30/75)	
Yes	47% (44/93)	
Neutropenia during ICU admission		0.535
No	47% (37/79)	
Yes	42% (37/89)	
Treatment limitations^b^		**< 0.001** *
No	37% (53/144)	
Yes	88% (21/24)	
ICU‐acquired infection		0.247
No	41% (46/113)	
Yes	51% (28/55)	

*Note*: Data are reported as the ICU mortality rate within each subgroup, with *p*‐values from Fisher's exact tests, unless stated otherwise. Bold *p*‐values are significant at *p* < 0.05.

Abbreviations: ICU, intensive care unit.

^a^ Ordinal/continuous variables were grouped into categories within which the ICU mortality rates are reported; *p*‐values are from Mann–Whitney *U* tests on the ungrouped data (e.g., comparing the time from hospital admission to ICU admission between patients that did vs. did not die in ICU).

^b^ At or within 24 h of ICU admission.

* Remains significant after adjustment for multiple comparisons using the Benjamini–Hochberg procedure (for *N* = 32 tests and a false discovery rate of 0.05).

**TABLE 5D:** Associations between organ failure in ICU and ICU mortality.

	ICU mortality rate	*p*‐Value
Level of organ failure^a^, ^b^		**< 0.001** *a
0	6% (1/18)	
1	20% (10/51)	
2	55% (26/47)	
> 2	71% (37/52)	
Respiratory failure		**< 0.001** *
No	7% (4/54)	
HFNO/NIV	52% (22/42)	
MV	67% (48/72)	
Cardiovascular failure		**0.020**
No	31% (17/55)	
Yes	50% (57/113)	
Gastrointestinal failure		0.350
No	45% (71/157)	
Yes	27% (3/11)	
Liver failure		0.138
No	42% (63/150)	
Yes	61% (11/18)	
Neurological failure		1.000
No	44% (71/162)	
Yes	50% (3/6)	
Renal failure		**< 0.001** *
No	33% (38/114)	
Yes	67% (36/54)	

*Note*: Data are reported as the ICU mortality rate within each subgroup, with *p*‐values from Fisher's exact tests, unless stated otherwise. Bold *p*‐values are significant at *p* < 0.05.

Abbreviations: HFNO, High Flow Nasal Oxygen; ICU, intensive care unit; MV, mechanical ventilation; NIV, non‐invasive ventilation.

^a^ Ordinal/continuous variables were grouped into categories within which the ICU mortality rates are reported; *p*‐values are from Mann–Whitney *U* tests on the ungrouped data (e.g., comparing the level of organ failure between patients that did vs. did not die in ICU).

^b^ The number of organs that failed during the ICU admission, considering a total of six: respiratory, cardiovascular, gastrointestinal, liver, neurological or renal.

* Remains significant after adjustment for multiple comparisons using the Benjamini–Hochberg procedure (for *N* = 32 tests and a false discovery rate of 0.05).

## 4 Discussion

This SE provides valuable insights into the characteristics and hospital outcomes of HM patients admitted to our ICU. As the first such evaluation locally, the results will inform future local practice and resource management and may encourage future national research initiatives, given the scarcity of UK‐based studies.

Our ICU (44.0%) and in‐hospital (53.0%) mortality rates were higher than the 29.4%–33.0% ICU and 45.7%–48.7% hospital mortality reported in other UK studies, and the 39.3% in‐hospital mortality reported in the largest to‐date relevant observational study [13, 14, 23]. This may reflect differences in patient populations, with our cohort showing higher admission illness severity, greater levels of organ failure, and longer ICU LOS than in comparable studies. It may also be explained by differences in inclusion criteria. Lower mortality studies may have included those admitted ‘with’, rather than ‘because of’ HM; for example, those in remission or unrelated presentations [23, 25]. In our SE, 22.2% of admissions were non‐HM‐related, most commonly unrelated surgeries or traumatic injuries, which were excluded from analysis (see Figure 1). A post hoc analysis showed a 12.5% (6/48) ICU mortality rate in non‐HM‐related ICU admissions, which was significantly lower than the 44.0% in those with HM‐related admissions (*p* < 0.001); pooling these two cohorts resulted in an ICU mortality rate of 37.0% (80/216), which is consistent with existing literature.

An average time to ICU admission of 10 days was seen, which could suggest a conservative local ICU admission policy, although several other explanations are plausible. A period of 10 days on the ward prior to ICU admission may also be characteristic of HM disease, where individuals were inpatients for ward‐based treatment prior to deteriorating and needing ICU admission, or where early admission to the ICU was not clinically required or appropriate in the context of disease trajectory. We also saw a high ICU mortality rate (88%) in patients where treatment limitations were set within 24 h of ICU admission. This would be expected, but also suggests there was an appreciation of a likely poor outcome given the timing of the decision, and could raise ethical debate as to whether exposing these patients to burdensome treatment in the ICU is beneficial for their QoL.

APACHE II, SOFA scores, chronic renal failure, HM‐subgroup, chemotherapy‐naïveté, time to ICU admission, SCT with GvHD, treatment limitations within 24 h of ICU admission, admission due to respiratory failure, degree of organ failure, requirement for vasoactive medication, advanced respiratory support and acute renal failure were found to be associated with increased ICU mortality. This aligns with previous literature demonstrating increased risk of mortality with vasopressor and MV requirement, but adds further inconsistency regarding illness severity scores, ICU admission timing, SCT, age, pre‐morbid frailty and remission status [22]. It is important to note the chemotherapy‐naïve group (*n* = 9) likely represents those with newly diagnosed aggressive diseases or those too unwell to undertake chemotherapy.

For the overall cohort, the ICU mortality rate in patients with a history of SCT was 43%, which was lower than that of some previous studies [23], although there is considerable variability in the literature [25]. However, dividing the analysis by the presence of adverse events found patients who did not develop GvHD post‐SCT had a lower ICU mortality rate than those that were CGT‐naïve (26% vs. 51%), whilst those developing GvHD had a considerably higher ICU mortality rate (70%). This may suggest that, whilst complication‐free SCT is protective, the development of GvHD is associated with a considerably higher likelihood of ICU mortality. Consequently, the between‐study variability in associations between SCT and ICU mortality may, in part, result from differences in rates of GvHD between studies. Further research in this area would be warranted to assess this hypothesis.

Our findings provide valuable insights for local practice. For example, the associations between admission APACHE II and SOFA scores and ICU mortality could be useful in facilitating early communication of expectations, allowing patients to engage in informed decisions regarding their care. Whilst these findings may support clinician decision‐making, until accurate and validated predictive models are created for HM patients, decision‐making should rely on regular re‐appraisal and clinician expertise [39]. It is plausible that there is opportunity to improve patient outcomes following this SE. For instance, if future, robust studies could confirm the observed association between longer times to ICU admission and mortality, this would suggest that changes to processes leading to earlier ICU admission (e.g., more proactive admission policies or detailed review of the initial ward‐based management in those patients who ultimately require ICU‐admission), may identify opportunities to improve patient care.

Several mobility‐ and rehabilitation‐related outcomes were also assessed. At ICU discharge, 31.9% of survivors were unable to mobilise out of bed (MMS ≤ 2), yet 94.9% of patients surviving to hospital discharge could actively mobilise (MMS ≥ 6). Patients may leave the ICU immobile because of ongoing medical issues (e.g., receiving vasoactive medication up to discharge) or due to sub‐optimal physiotherapy input (limited resources, absent weekend rehabilitation services or being de‐prioritised based on risk [40]). In addition, patients experienced a relatively short ICU LOS (median: 6 days) compared to their overall hospital LOS (30 days). Prolonged HM‐ or critical illness‐related treatment may explain the ward LOS and mobility status, and it is probable that the improvements in MMS scores from ICU to hospital discharge are driven predominantly by recovery from acute illness, with patients regaining mobility once health improved. Prolonged ward LOS may be a consequence of the time taken to rehabilitate physical disability. However, detailed data relating to physiotherapy intervention, including dosing, timing and intensity which would be required to assess this were not collected as part of this SE. Consequently, this remains an area that warrants further investigation in a future study.

Of patients surviving to hospital discharge, 25.3% required ongoing rehabilitation, suggesting persisting disability [2], with the estimated 12‐month post‐hospital discharge survival being 72%. Patients with poorer performance status are less likely to be offered further adjunctive treatment such as chemo/radiotherapy or to be re‐admitted to the ICU [20, 23]. Whether enhanced physiotherapy can elicit improved functional outcomes is an important avenue for future research, as this may directly lead to improved hospital outcomes, health‐related QoL and life expectancy through the continued access to adjunctive life‐sustaining therapy [28].

It is important to understand the degree to which physiotherapy input modifies functional outcome. There is a complex interaction between natural recovery, clinical factors limiting rehabilitation progress, and the changes induced by physiotherapy treatment in this HM population. Case‐note review and prospective studies may help to unravel this, which would then support the optimal allocation of stretched therapy resources to those most likely to benefit, at the most beneficial time. It may also identify whether functional outcomes are independently associated with overall hospital outcomes, which was not explored in this SE. Future iterations of this work should seek to identify the patients most likely to benefit from input, as well as identifying the optimum time for physical rehabilitation interventions, guiding the creation of ethical resource allocation frameworks.

### 4.1 Strengths and Limitations

This SE had several strengths, chief amongst which is the fact that it represents the first time that outcomes of HM patients admitted to one of the UK's largest ICUs have been comprehensively described. The SE also included complete datasets for all patients for a wide range of relevant factors and outcomes. However, there are also some limitations that need to be considered when interpreting the results. Primarily, this was a single‐centre SE, meaning that results may not be generalisable to other centres. This is particularly pertinent given the size, speciality and individualised ICU admission policy of our centre, which may not be reflective of other centres. Second, the SE design also precluded performing multivariable or adjusted analyses; hence, findings may be subject to confounding, further limiting generalisability. Third, whilst the sample size was comparable to other studies in the literature, it was still relatively modest. As such, the analyses assessing associations with ICU mortality only had moderate statistical power, meaning that only relatively large effect sizes were detectable, and increasing the risk of false negatives. Fourth, since over 30 factors were considered when analysing associations with ICU mortality rates, it is possible that some statistically significant findings may represent false positives, particularly those that did not remain significant at the Benjamini–Hochberg‐adjusted significance threshold (as indicated in the tables). Finally, whilst mobility and physical function was an interest of the SE, comprehensive analysis of physiotherapy input throughout admission was not collected.

## 5 Conclusion

This SE comprehensively describes the characteristics and hospital outcomes of patients admitted to our ICU because of HM. Several factors associated with ICU mortality were identified, including APACHE II and SOFA scores, as well as acute renal, cardiovascular and respiratory failure. Understanding these factors may enhance local understanding to guide future practice and potentially lead to improvements in patient care. Physical outcomes and physiotherapy interventions should be analysed in detail to identify whether functional outcomes are modifiable in critically ill HM patients and whether optimised input is possible. Further multi‐centre prospective studies are warranted, to validate the findings of this SE.

## Author Contributions

**Adam Harriman**: conceptualisation, methodology, analysis, investigation, writing – original and review, visualisation. **Dhruv Parekh**: supervision, project administration. **Jonathan Weblin**: supervision, writing – review and editing. **James Hodson**: methodology, formal analysis, writing – review and editing. **Vicky MacBean**: supervision, conceptualisation, methodology, writing – review and editing.

## Funding

The authors have nothing to report.

## Ethics Statement

This project was registered and approved locally as a service evaluation and therefore, formal ethical approval was not required.

## Consent

The authors have nothing to report.

## Conflicts of Interest

The authors declare no conflicts of interest.

## Data Availability

The data supporting the findings of this service evaluation are available on request from the corresponding author only. The data are not publicly available due to privacy and ethical restrictions.
